# Suitability Assessment of Legal Regulation of Chemical Concentrations According to Vapor Pressure and Damage Radius

**DOI:** 10.3390/ijerph16030347

**Published:** 2019-01-26

**Authors:** Hyo Eun Lee, Seok J. Yoon, Jong-Ryeul Sohn, Da-An Huh, Seok-Won Jang, Kyong Whan Moon

**Affiliations:** 1 Department of Health Science, Korea University, Anam-ro 145, Seongbuk-gu, Seoul 02841, Korea; chokbab@naver.com (H.E.L.); ehslab@naver.com (S.J.Y.); sohn1956@korea.ac.kr (J.-R.S.); black1388@korea.ac.kr (D.-A.H.); 2 Environmental Research Complex, National Institute of Environmental Research, Hwangyeong-ro 42 Seo-gu, Incheon 22689, Korea; jsssws100@korea.kr

**Keywords:** Chemicals Control Act, concentration of chemicals, vapor pressure, Areal Location of Hazardous Atmospheres (ALOHA), Process Hazard Analysis Software Tool (PHAST), RMP*Comp, Korea Off-site Risk Assessment Supporting Tool (KORA)

## Abstract

Many chemicals used in the industrial field present risks, which differ depending on their chemical properties. Additionally, their various physicochemical properties change considerably with concentration. Many chemicals are used in customized processes in factories in the form of different aqueous solutions. The Korean Chemicals Control Act evaluates “hazardous chemicals”, describes their risks to the public, and regulates their concentration. To prepare against chemical accidents, factories construct models of potential damage radius, which is greatly influenced by a chemical’s vapor pressure. This study selected substances with widely varying vapor pressures (hydrogen fluoride, hydrogen chloride, aqueous ammonia, and hydrogen peroxide) and compared the results of different modeling programs (KORA, ALOHA, PHAST, and RMP*Comp) for various aqueous solution concentrations. The results showed that damage radius and vapor pressure increased similarly for each substance. Damage radius was negligible at low concentrations for all substances studied. Damage radius of ammonia solution increased with vapor pressure. Hydrogen fluoride is not found in aqueous solution at concentrations of less than 37%, and hydrogen peroxide does not show a large damage radius at low concentrations. However, the Chemicals Control Act strictly regulates hydrogen fluoride concentration beginning at 1%, hydrogen chloride and aqueous ammonia at 10%, and hydrogen peroxide at 6%. To effectively prepare against chemical accidents, we must examine scientifically-based, suitable regulations based on physicochemical properties.

## 1. Introduction

Chemicals have a direct and immediate impact on human life. The progress of science and technology nowadays necessitates the use of chemicals in product manufacturing and use. In Korea, approximately 43,000 kinds of chemical substances are distributed, and 418 million tons of chemical substances were used in 2016 [1]. Chemical substances used in the industrial field must be managed at the national level, because the raw materials themselves present risks associated with chemical substances. In the Korean chemical industry, shipments reached 119 trillion won (USD 103.5 billion), the seventh largest value in the world (as of 2013), and chemical production amounted to 187 trillion won, accounting for 16.7% of the manufacturing industry overall [2]. In response to the 2012 Gumi Hydrogen Fluoride Accident, the Korean Ministry of the Environment established the Chemicals Control Act in 2015. Laws to govern chemical substance management existed prior to this act, however, the Chemicals Control Act regulates all facilities involved in the process, from raw materials to products. In addition to the Chemicals Control Act, which is intended to prepare against environmental and chemical accidents, the Occupation Safety and Health Act, enacted by the Ministry of Employment and Labor, incorporates a Process Safety Management (PSM) plan that establishes and regulates internal industry guidelines for safe operation and work [3].

Both sets of regulations are based on similar laws in the United States: the Risk Management Plan (RMP) of the Environmental Protection Agency (EPA), and the PSM system of the Occupational Safety and Health Administration (OSHA). The US’s PSM system is intended to protect workers in factories, and thus involves change management, maintenance, caution, and process risk analysis. In contrast, the RMP is primarily intended to protect residents and the local environment, and thus involves procedures to cooperate with local fire departments and police stations and to cope with chemical accidents. The RMP also includes preventive programs, emergency response programs, and information disclosure [4].

The American and Korean systems of chemical management are roughly similar; their one main difference concerns concentration of the regulated chemical substances. In the US, the RMP and PSM regulate the amount of chemicals used. Chemicals are subject to management depending on how much of the chemical is used in the workplace. Only a few substances are subject to concentration regulation. The RMP regulates hydrochloric acid, for example, at concentrations of 50% or greater [5]. Aqueous ammonia is regulated at concentrations of 20% or greater by the RMP and 44% or greater by PSM [6]. In contrast, Korea’s Chemicals Control Act regulates both hydrochloric acid and aqueous ammonia at 10%, a remarkably low concentration. The act also specifies regulated concentrations for all 837 substances designated as “hazardous” and 97 substances that require preparation for chemical accidents [3]. In the US, there is concentration regulation only for some chemicals (hydrogen chloride, hydrogen fluoride, aqueous ammonia and hydrogen peroxide) [7]. The substances are toxic chemicals, but their vapor pressure differs greatly with concentration [8,9].

Specific concentrations of these chemicals are used commonly in industrial production. In Korea, the law requires an annual report on the use of hazardous chemical substances in workplaces. According to the results of the 2017 report, hydrogen fluoride is mainly used at a concentration of 50% to 60%, hydrogen chloride at 30% to 40%, aqueous ammonia at 20% to 30%, and hydrogen peroxide at 35%. This demonstrates a considerable difference from the regulation level of the Chemicals Control Act [10].

The differences between countries in regulated concentration of chemical substances also suggests a serious problem. Countries with low concentrations of chemical regulations may have excessive regulation. Conversely, countries with high concentrations of chemical regulations can’t manage low-concentration chemicals, so chemical accidents may occur in low concentrations. It is significant that regulatory standards differ according to the applicable laws and regulations. For example, PSM and the RMP differ in purpose. PSM prepares against chemical accident within the industrial field, whereas the RMP protects against chemical accidents outside the workplace. However, both are intended to prevent chemical accidents. All legal regulations should be based on scientific evidence and realistic regulations for each national industry.

This study used modeling programs to measure values according to the toxic concentration Emergency Response Planning Guideline (ERPG-2) [11] and the Acute Exposure Guideline Levels (AEGL-2) [12] for various concentrations of chemical substance, and estimate each modeling program to obtain damage radius. The modeling programs used included RMP*Comp [13], a free program distributed by the EPA; Areal Location of Hazardous Atmospheres (ALOHA) [14], developed for the U.S. EPA by the National Oceanic and Atmospheric Administration (NOAA); the Korea Off-site Risk Assessment Supporting Tool (KORA) [15], distributed by the Korean Ministry of Environment; and the Process Hazard Analysis Software Tool (PHAST) [16], which is widely used for commercial purposes. Damage radius was computed for each concentration of hydrogen chloride, hydrogen fluoride, aqueous ammonia, and hydrogen peroxide. This study’s objective is to compare and analyze Korea’s legal regulations and predicted damage radius in case of a chemical accident.

## 2. Materials and Methods

### 2.1. Selection of Chemical Substances

This study selected as subjects chemical substances that are used in many industries, that differ widely in vapor pressure, that have similar physicochemical properties as those commonly included in the RMP, PSM, and the Chemicals Control Act, and that have specific concentrations. These substances are used in industrial settings, especially in the form of aqueous solutions [17]. The representative subjects selected were hydrogen fluoride, hydrogen chloride, hydrogen peroxide, and aqueous ammonia. The basic physical and chemical properties of each substance are shown in Table 1 [18].

### 2.2. Legal Regulation of Chemical Substances

Each selected chemical is characterized by an irritating odor and smell; some are strongly acidic and used for sterilization and disinfection. In Korea, they are controlled by the Chemicals Control Act. A “toxic substance” is a harmful chemical substance, designated by the Minister of Environment according to standards set by Presidential Decree, the hazards of which are determined annually by the National Institute of Environmental Research [3]. “Chemicals requiring preparation for accidents” are those designated and disclosed by the Minister of Environment as likely to cause chemical accidents due to strong acute toxicity or explosiveness [3]. In the U.S., facilities that store or use chemical substances identified by OSHA as toxic and reactive must submit to PSM [5]. The EPA Code of Federal Regulations, Title 40, Part 68 outlines chemical accident preparedness, countermeasures, and the RMP.

Globally, chemicals are managed independently by each country. Relevant chemical substance regulations in several countries are presented in Table 2. Notably, base-level concentrations differ across each country. In Korea specifically, the concentrations for hydrogen peroxide are significantly different even between PSM and the Chemicals Control Act. The regulatory concentrations of the U.S. are generally high compared to those of the three East Asian countries, and the E.U. does not specify any regulatory concentration at all.

### 2.3. Amount of Chemicals in Industry

The Chemicals Control Act requires an annual report on the concentration, usage, and purpose (e.g., cleaning agent, plating solution, pH adjuster, etc.) of toxic chemicals used in Korea [3]. The amount of each substance used according to the 2017 report is shown in Table 3. The data show that chemical substances are primarily used at specific concentration, and there is a considerable difference between the actual concentrations used in practice and the regulated concentrations. Especially, Korea is a country where engineering industry and manufacturing have developed, and it would be similar situation if there is a similar industrial form [10].

As of 2017, more than 400 of the industrial sites surveyed use aqueous ammonia at a concentration of 20% to 30%, most commonly 25%. Hydrogen chloride is used in concentrations of 30% to 40%, most commonly 35% or 36%. Hydrogen fluoride is used in concentrations of 50% to 60%, most commonly 55%. Finally, hydrogen peroxide is used in concentrations of 30% to 40%, most commonly 35%. Most low-concentration chemicals are handled in small containers, such as laboratory containers and 20 L or 200 L drums, whereas high-concentration chemicals are handled in bulk tanks and tanker trucks.

### 2.4. Physicochemical Properties (Vapor Pressure)

Chemical substances have many physicochemical properties. With respect to chemical accidents, the characteristic most relevant to the chemical’s potential to spread and damage the surrounding area and environment is vapor pressure or volatility. The higher the vapor pressure, the smaller the attractive force between the molecules, which means the fluidity of the molecules increases and the vaporization is better. The higher the vapor pressure, the more vaporized and spreads away. [19] Whether in liquid or gas form, chemicals with a high vapor pressure experience very high vaporization following a leak and a rapid rate of diffusion. Table 4 shows the vapor pressures at various concentrations for the four chemical substances studied. Hydrogen fluoride does not exist in a liquid state at low concentration. Hydrogen peroxide has a large difference in vapor pressure compared to other chemical substances, but the absolute value of its vapor pressure is not large [9,20].

### 2.5. Setting Concentration of Interest

Concentration guidelines for exposure predict how people exposed to certain hazardous chemicals will be affected in an emergency response situation. The Emergency Response Planning Guidelines (ERPGs) and Acute Exposure Guideline Levels (AEGLs) are the measures most commonly used by both the EPA in the U.S. and the Ministry of Environment in Korea. These guidelines use a three-category structure to rank exposure values for the chemicals to which they are applied; individual values are specific to each chemical concentration, but the three categories are similar. A level 1 value indicates temporary damage, a level 2 value indicates failure or serious health effects, and a level 3 value indicates life-threatening effects. The EPA’s ALOHA preferentially applies AEGL and ERPG values in modeling. KORA applies ERPG and AEGL values first. ERPG-2 and AEGL-2, which represent the intermediate exposure levels, were selected as the application concentrations for modeling in the present study.

#### 2.5.1. ERPG-2

The ERPGs were developed by the Emergency Response Planning Committee of the American Industrial Hygiene Association (AIHA). Concentrations are set for over 150 chemicals with corresponding estimates of the health effects to most people after one hour of airborne exposure. The ERPGs do not include elderly people, those with existing health issues, or children in their estimates. ERPG values are not used to measure AEGL, but to protect the public in the event of chemical leaks over a short duration (one hour). The values should not be used to evaluate risk to workers or cases of long-term exposure:

- ERPG-3 indicates the maximum airborne concentrations to which individuals may be exposed for up to one hour without experiencing or developing life-threatening health effects or symptoms.
- ERPG-2 indicates the maximum airborne concentrations to which individuals may be exposed for up to one hour without experiencing or developing irreversible or other serious health effects or symptoms.
- ERPG-1 indicates the maximum airborne concentrations to which individuals may be exposed for up to one hour without experiencing more than mild, transient adverse health effects, or without perceiving a clearly discernable objectionable odor [11].

#### 2.5.2. AEGL-2

The AEGLs suggest that most people, including vulnerable groups such as elderly people, those with existing health issues, and children, will begin to experience health effects after a certain period of exposure to chemicals. Whereas ERPG values represent varying effects of concentrations after a standard one-hour exposure, AEGL values represent allowable concentrations for various periods of time (10 min, 30 min, 60 min, four hours, and eight hours). This is more easily determined. The AEGLs were initially developed by the National Advisory Committee for AEGLs, and are now maintained and further developed by the National Academy of Sciences. Standards are currently set for 175 chemicals:

- AEGL-3 indicates the airborne concentration above which the general population, including vulnerable groups, may experience life-threatening health effects or mortality.
- AEGL-2 indicates the airborne concentration above which the general population, including vulnerable groups, may experience irreversible or serious and long-lasting adverse health effects, or an impaired ability to escape the area.
- AEGL-1 indicates the airborne concentration above which the general population, including vulnerable groups, may experience discomfort, irritation, or certain asymptomatic and non-sensory effects that are non-disabling, transient, and reversible upon cessation of exposure [12].

### 2.6. Damage Radius Estimation Method

Assuming all leaks occur under the same conditions, saturation vapor pressure differs for each concentration; concentration and saturation vapor pressure can thus be modified in a modeling program. KORA automatically sets vapor pressure for each substance and determines its concentration.

For modeling, concentrations of 10% to 42% were used for hydrochloric acid, and concentrations of 37% to 70% for hydrofluoric acid. Aqueous ammonia can only be used in concentrations of 1% to 30%; hydrogen peroxide can be used in all concentrations. On the other hand, in the modeling program COMP used in the U.S. RMP, there are only a few limitations of the reference concentration, and in the case of ALOHA, the FACTOR value can be modified, but the default value is calculated as 100%.

The damage radius, which is a key part of this study, was derived from the concentrations of toxic substances that reached when they leaked under the same operating conditions, depending on the concentration of each substance in the study. Because the modeling is carried out based on the vapor pressure, the method of deriving the influence range proceeded under controlled conditions.

All the modeling programs (KORA, ALOHA, PHAST) are based on the Gaussian model an atmospheric diffusion model [21]. The Gaussian air diffusion model is currently the most widely used model for environmental impact assessment. It is assumed that the leak occurs at one point, and no chemical reaction occurs during the leak. It is also assumed that the leak rate is constant. This characteristic is consistent with the modeling theory assuming that the chemical forms a pool and evaporates and diffuses continuously due to the vapor pressure. However, the modeling program calibrates itself with respect to diffusion and produces the results. For example, Gaussian modeling is performed at low concentration, but it is converted to a SLAB model (heavy gas diffusion model) at high concentrations of aqueous solutions. 

#### 2.6.1. Modeling Program Selection

Four modeling programs were selected. ALOHA, supported by the EPA, has the advantages of allowing the user to select an aqueous solution and to input desired data values. The program is easy, quick, and free, and thus popular among many users. However, it does not simulate atmospheric chemical reactions, and it cannot calculate three-dimensional concentration distribution.

KORA, supported by the Korean Ministry of Environment, is a free program produced and distributed by the government to support compliance with the legal regulations of the Chemicals Control Act. Due to continual updating and strengthening of chemical management laws, the tool undergoes continuous improvement, but it presents a disadvantage in that desired data values cannot be input for modeling, as in ALOHA. However, it enables users to model leakage of an aqueous chemical solution and to quickly report the results.

PHAST, developed by Det Norske Veritas (DNV), a joint venture between Germany and Norway, is globally recognized as a quantitative risk assessment tool that can model not only pure substances but also mixtures. Additionally, it realistically models atmospheric chemical reactions following a leak. However, due to high cost and complex operation, its availability and accessibility are limited [22].

#### 2.6.2. Weather Conditions (Wind Speed, Atmospheric Stability)

To isolate and confirm the concentration of the studied chemical substances, we standardized and set all other modeling conditions according to the Technical Guidelines for the Selection of Accident Scenarios, provided by the Chemical Safety Authority of the Korean Ministry of Environment [23]. Based on these guidelines, stable atmospheric conditions are identified as *F* in Table 5. The ambient temperature is 25 °C and the atmospheric humidity is 50%. The wind speed is estimated at 1.5 m/s.

#### 2.6.3. Endpoint Concentration

The endpoint concentration is the distance that the concentration reaches. That is, for example, the distance that 50 ppm reaches varies depending on the concentration of the aqueous solution. The Korea Chemicals Control Act adopts the ERPG-2 value designated by AIHA as the endpoint concentration. If an ERPG-2 value is not available, the AEGL-2 value is adopted. Chemicals with neither ERPG-2 nor AEGL-2 values may adopt the PAC-2 value published by the US Department of Energy (DOE) [23]. The present study calculated the damage radius of each chemical substance by setting the ERPG-2 and AEGL-2 endpoint concentrations (Table 6) as the concentration of interest.

#### 2.6.4. Leakage and Leak Scenario Estimation

Chemicals in liquid form at atmospheric temperature were assumed to leak out in 10 min to form a liquid layer. The tank containing the chemical substance was assumed to have a storage capacity of 5000 kg. The liquid layer was assumed to be 1 cm deep when formed in the absence of diffusion preventive measures, such as a discharge wall. These parameters are in accordance with the EPA’s RMP manual [24]. The rate of evaporation and diffusion into the atmosphere was calculated as follows [23]:

(1) $$R_{E}=\frac{1.4\times U^{0.78}\times M_{w}{}^{\frac{2}{3}}\times A\times P_{v}}{82.05\times T}$$

where *U* is wind speed (m/s), *M_W_* is molecular weight, *A* is the surface area of the liquid layer (m^2^), *P_V_* is vapor pressure (mmHg), and *T* is temperature (K). The vapor pressure according to chemical concentration is calculated based on the values in Table 4.

The surface area *A* of the liquid layer the surface area formula is as follows:

(2) $$A=0.1\times \frac{Q}{P}$$

where *A* is surface area of the liquid layer (m^2^), *Q* is quantity (kg), and *P* is density (kg/m^3^). However, when a discharge wall is used, the area of the discharge wall and the surface area of the liquid layer are smaller. Based on a leakage amount of 5000 kg, the surface area of the liquid layer was derived for each chemical substance [23].

## 3. Results

### 3.1. Damage Radius by Chemical Substance

The damage radiuses calculated under the same conditions by each modeling program are presented below; Table 7 shows the results for hydrogen fluoride. The toxicological value specified by ERPG-2 for the subject chemicals is higher than that of AEGL-2. Consequently, damage radiuses calculated using the ERPG-2 values were larger than those using AEGL-2 values in all modeling programs. In RMP*Comp, modeling is only possible for certain concentrations. In PHAST, a 70% concentration of hydrogen fluoride could not be modeled. Each modeling program produced different specific results, but all showed that damage radius increased considerably along with concentration. In general, ALOHA produced the largest damage radii.

Our analysis also compared the damage radiuses calculated at the lowest and highest concentrations for each chemical. For hydrogen fluoride, KORA’s predicted damage radius for the highest concentration was approximately five times greater than that of the lowest; PHAST’s was three times greater; and ALOHA’s was 10 times greater.

Aqueous solutions of hydrogen chloride (Table 8) showed a high damage radius for concentrations of 35% and higher. Considerable differences were also observed across modeling programs. The damage radius predicted by KORA’s highest concentration was approximately 200 times greater than the lowest; ALOHA’s was seven times greater; and PHAST’ 100 times greater. In RPM*Comp, modeling was enabled only for certain concentrations, namely 37% and 38%, because these are the concentrations of hydrogen chloride typically used for industrial purposes.

Hydrogen peroxide (Table 9) can be prepared in concentrations from 5% to 100%. Its vapor pressure is not particularly large relative to that of the other chemical substances. However, it is designated as a “Chemical requiring preparation for accidents” by the Chemicals Control Act and is subject to PSM. ERPG-2 and PAC-2 concentration values are the same for hydrogen peroxide at 50 ppm. According to the KORA modeling results, hydrogen peroxide appears to be a low-risk substance; however, the ALOHA and PHAST models both showed a wide damage radius. The only three chemical modeling programs are those with similar minimum and maximum concentration differences. Damage radius was found to be no more than two times greater at the lowest and highest concentrations in all modeling programs. The PHAST results showed that damage radius increases up to a concentration of 80%, then decreases from concentrations of 85% to 100%. RMP*Comp does not currently support modeling for hydrogen peroxide.

For aqueous ammonia (Table 10), damage radius increased constantly with concentration. Of all the modeling programs, ALOHA showed the most widespread toxic results. RMP*Comp supports modeling only at certain concentrations. The damage radius predicted by KORA for the highest concentration was approximately 10 times greater than the lowest; ALOHA’s was approximately 6.5 times greater; and PHAST’s was approximately 5.6 times greater, showing no considerable difference between the lowest and highest concentrations across the modeling programs.

### 3.2. Comparison of Damage Radius of Common Industry Use Concentration and Regulatory Concentration

For each substance, we compared the damage radius of the legally regulated concentration and the concentration most commonly used in industry. The numerical values in Table 11 express this comparison, assuming the damage radius of the regulatory level to be 1. The greater the value, the greater the difference between the legal regulation level and the level commonly used in the industrial field. Although hydrogen fluoride is regulated at a concentration of 1%, it is not possible to model concentrations this low; thus, hydrogen fluoride was compared based on the damage radius of a 37% concentration, the lowest supported by modeling. Similarly, hydrogen chloride was calculated on the basis of the model able 20%.

### 3.3. Correlation between Physicochemical Properties and Damage Radius

**Figure 1:**
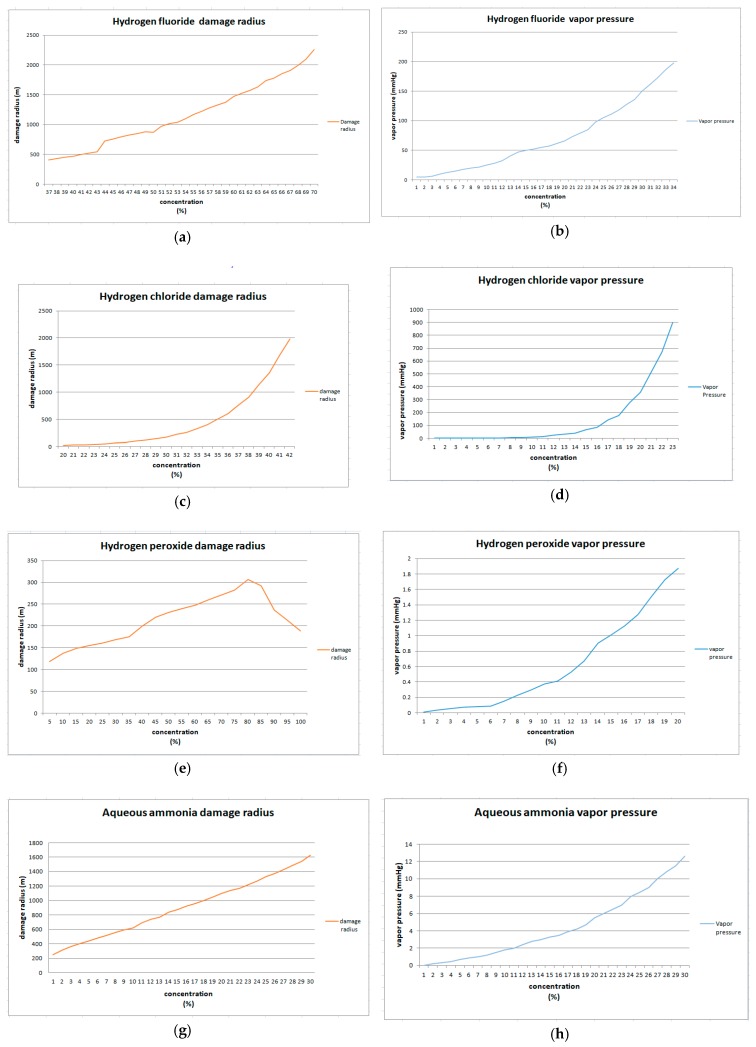
Comparison of damage radius and vapor pressure for each chemical substance studied. In each graph, the horizontal axis represents the concentration (%) range of the substance, and the vertical axis represents the damage radius (m) and vapor pressure (mmHg): (**a**) Hydrogen fluoride damage radius; (**b**) Hydrogen fluoride vapor pressure; (**c**) Hydrogen chloride damage radius; (**d**) Hydrogen chloride vapor pressure; (**e**) Hydrogen peroxide damage radius; (**f**) Hydrogen peroxide vapor pressure; (**g**) Aqueous ammonia damage radius; (**h**) Aqueous ammonia vapor pressure.

The purpose of this study was to investigate the relationship between the damage radius and the physicochemical properties (specifically vapor pressure) of the chemical substances selected. This is because the physicochemical properties of a chemical must be considered when setting its regulated concentration. Damage radius was calculated by averaging the results obtained by modeling programs. For all the chemicals studied (except for hydrogen peroxide at concentrations of 85% to 100%), damage radius increased along with concentration. Significant increases in damage radius at specific concentrations were compared by curving vapor pressure and damage radius (Figure 1).

Hydrogen fluoride shows a small damage radius at low concentrations, but rapidly increases at concentrations of 50% or more. The damage radius of hydrogen chloride increases sharply at concentrations above 35%. In the case of hydrogen peroxide, vapor pressure increases along with concentration, but diffusion slows due to increase of the mole fraction during modeling. The damage radius of aqueous ammonia increases at a constant rate with concentration, but is seven times greater in 30% concentrations than in 1% concentrations. This indicates a significant correlation between vapor pressure and damage radius.

### 3.4. Statistical Analysis of Chemical Substances and Vapor Pressure

**Figure 2:**
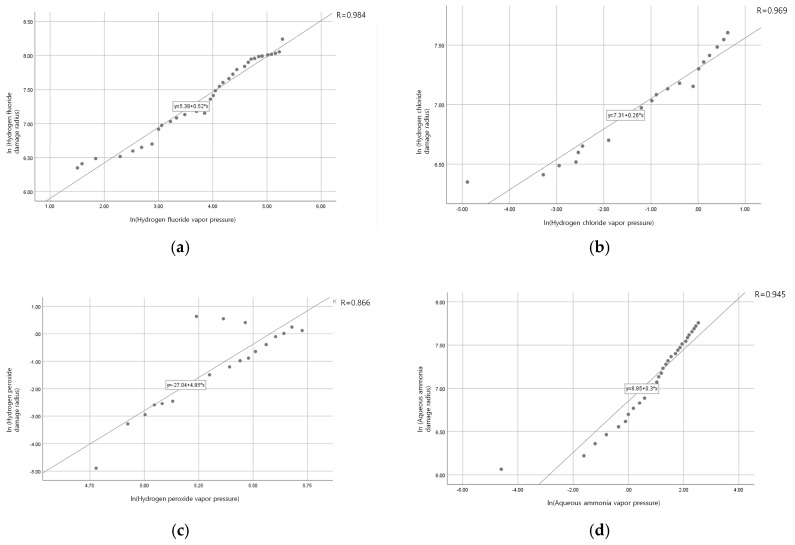
Correlation between damage radius and vapor pressure for each substance studied: (**a**) Hydrogen fluoride; (**b**) Hydrogen chloride; (**c**) Hydrogen peroxide; (**d**) Aqueous ammonia.

The relationship between vapor pressure and damage radius was analyzed for each chemical substance using the Pearson correlation coefficient (Figure 2). In contrast to Spearman correlation analysis, which is applied to categorical variables, Pearson analysis is applied to continuous variables [25] All data were analyzed using IBM SPSS Statistics 25 (SPSS Inc., Chicago, IL, USA). The significance level for all statistical analyses was set at *p* < 0.05. Correlations resulting from statistical analysis are indicated by the *R*-value, expressed as a numerical value between −1 and 1. A negative *R*-value indicates negative correlation. Values between −1 and −0.7 indicate strong negative correlation. Values of −0.7 to −0.3 indicate normal negative correlation. Values between −0.3 and 0 indicate weak negative correlation. Similarly, a positive *R*-value indicates positive correlation. The closer the *R*-value is to 1, the stronger the positive correlation; the closer to 0, the weaker the correlation [26].

Hydrogen fluoride demonstrated the strongest correlation between vapor pressure and damage radius, followed by hydrogen chloride, aqueous ammonia, and hydrogen peroxide. This indicates that the predicted damage radius of a chemical accident is strongly related to the chemical’s vapor pressure. Thus, it is necessary to give great weight to the physicochemical characteristic of vapor pressure when setting the regulated concentration of a chemical substance.

## 4. Discussion

This study investigates the appropriateness of the chemical concentration regulations in Korea’s Chemicals Control Act. Appropriate regulation of chemical substance concentration is important, because if a product’s content exceeds the regulated concentration, the product is regulated as a toxic substance. For example, if 5000 kg of product is manufactured with a concentration of 11% hydrogen chloride, which exceeds the regulated concentration of 10%, the regulation is enforced by calculating the damage radius for 5000 kg of product containing 100% hydrogen chloride. Overly strict regulations may thus subject factories to disadvantages.

### 4.1. Hydrogen Fluoride

In the case of hydrogen fluoride, any concentration exceeding 1% is regulated as a toxic chemical. As shown by the ALOHA modeling results, hydrogen fluoride can vary widely in concentration. The damage radius of the highest concentrations was about five times greater than the lowest concentration in KORA, 10 times greater in ALOHA, and three times greater in PHAST. For concentrations of hydrogen fluoride over 65%, some increase and decrease in damage radius was observed, although the phenomenon occurred only in the KORA results. Because hydrogen fluoride exhibits a rapid increase in damage radius at higher concentrations, it has the potential to cause dangerous chemical accidents. However, because actual hydrogen fluoride solutions exist only in the range of 37% to 70% concentration, the regulation of 1% concentration for hydrogen fluoride is extreme. The concentration of hydrogen fluoride most commonly used in industrial fields is 55%. Korea, Japan, and China regulate hydrogen fluoride concentration at between 1% and 5%, but it is not possible to model concentrations in this range. All the modeling programs used (KORA, PHAST, and ALOHA) showed about a threefold difference in damage radius between the industry standard concentration of 55% and the lowest concentration that can be modeled, 37%. This indicates that the regulated concentration standard is highly inconsistent with the reality of use at industrial sites.

### 4.2. Hydrogen Chloride

Hydrogen chloride solution begins to show a high damage radius around a concentration of 35%. At 20%, damage radius shows a slight increase; when the concentration reaches 35%, it increases sharply. The RMP*Comp support program supports modeling only of 37% or 38% aqueous solutions. Like hydrogen fluoride, the damage radius of hydrogen chloride varies greatly by concentration. In KORA, the damage radius was 200 times greater at the highest concentration than the lowest, in ALOHA it was 100 times greater, and in PHAST it was seven times greater. We additionally attempted to compare the damage radius of hydrogen chloride at the regulated concentration and industry standard concentration; however, it is not possible to model the regulated 10% concentration. The damage radius of the industry standard concentration was 50 times greater than that of the lowest concentration that can be modeled (20%) in PHAST, and three times greater in ALOHA. Because hydrogen chloride is present in aqueous solution only at 20% concentration or greater, it is reasonable to set the regulated concentration close to 20%, even calculating conservatively. There is a scientific basis to set the regulated concentration in the vicinity of 35% to safeguard against chemical accidents.

### 4.3. Hydrogen Peroxide

Hydrogen peroxide is the only substance that can be modeled at full concentration. The absolute value of its vapor pressure is small compared to that of the other chemical substances studied. The KORA results showed an overall damage radius of less than 50 m for hydrogen peroxide. However, the ALOHA and PHAST results showed a broader radius, exceeding 150 m. Hydrogen peroxide is not targeted by the RMP, nor is modeling supported in RMP*Comp. The PHAST results showed a tendency for damage radius to decrease at concentrations of 85% and above. In this modeling program, the higher the concentration of the substance, the heavier the steam becomes, making diffusion more difficult. In the Chemicals Control Act, hydrogen peroxide has different regulations for the categories of “Chemicals requiring preparation for accidents” (35%) and “toxic chemicals” (6%). Additionally, the PSM regulated concentration for hydrogen peroxide is 52%. Multiple regulated concentrations can also be problematic. To fulfill the purpose of chemical accident prevention, regulated concentrations must be consistent.

### 4.4. Aqueous Ammonia

Aqueous ammonia may be found in concentrations of 10%, the nationally regulated level. The damage radius of a 25% concentration, which is most commonly used in industry, is about two times greater than the regulated standard. The appropriate regulatory concentration of aqueous ammonia requires ongoing research and experimentation.

In all cases, the regulated concentrations of the chemicals studied and the range of concentrations mainly used in industry are very different. To select a reasonable concentration for regulation, it is important to consider not only the concentrations of the chemical substance used most commonly in industry, but also the chemical’s physicochemical properties. This study illustrates that among the various physicochemical characteristics, vapor pressure is particularly important in considering regulatory concentrations of chemical substances.

### 4.5. Overall

Currently, the Korean Chemicals Control Act designates “toxic substance” according to the declarations of the National Institute of Environmental Research (NIER) [27,28]. NIER makes its declarations based on information about the chemical substances’ toxicity. The Chemical Safety Authority also designates and specifies substances (chemicals requiring preparation for accidents) liable to cause accidents, distribution quantities for chemicals in Korea, and chemical properties with the potential to cause accidents (e.g., flammability). However, chemical accidents can occur with any chemical, and the potential damage cannot be predicted by toxicity or physicochemical properties alone. It is problematic that each individual country independently sets legal regulations for the concentration of chemical substances. In the EU, no regulated concentration is specified for harmful chemical substances, and there is a possibility that the chemical substance to be treated at a very low concentration is also a legal object. In the three East Asian countries of Japan, Korea, and China, compliance with the legal regulations may be difficult in the industrial field because the regulations are not consistent with reality. Furthermore, even if a regulated concentration is set, chemical substances may post other risks depending on their physicochemical properties. For example, it is meaningless to set a concentration for flammable substances, which can cause a fire at any concentration depending on properties such as flash point, upper limit of explosion, and so on [29]. The results of this study indicate that the appropriateness of legal regulations regarding concentration of chemical substances should be examined based on the three collective aspects of concentration, reality of use in the industrial field, and likelihood of chemical accidents.

## 5. Conclusions

This study examines the adequacy of the legal concentration regulations in Korea’s Chemicals Control Act. Specifically, the legal regulations were evaluated according to various concentrations of chemicals with a range of vapor pressures. The results showed a considerable difference between predicted damage radius at the regulated concentration and at the concentration typically used in the industrial field. Hydrogen fluoride and hydrogen chloride in particular could not be modeled as aqueous solutions at regulated concentration. Additionally, a high correlation was found between vapor pressure and the damage radius by chemical concentration. This shows that reasonable legal regulations can be set by considering the physicochemical property of vapor pressure.

Compared with those of other countries, the concentration regulations of Korea’s Chemicals Control Act and other East Asian countries are very strict (Table 2). Korea’s regulations are similar to those of Japan, though for a different purpose: Japan’s regulations are intended to control not chemical accidents, but rather workers’ exposure levels. In the U.S., which does regulate concentrations in PSM or the RMP to control chemical accidents, the regulations are much more relaxed than those in Korea.

The U.S. also regulates chemical concentrations differently to control workplace exposure levels and chemical accidents. Worker exposure is regulated according to a time-weighted average (TWA), based on frequent exposure for over 8 h. Regulations to prepare against chemical accidents include PSM and the RMP, which specify concentrations significantly higher than those for the TWA [30]. In Korea, however, the Chemicals Control Act regulates workplace personal protective equipment and material safety data sheets (MSDS) based on a single concentration. Given this, the adequacy of the regulatory concentrations to control chemical accident and worker exposure levels should be carefully considered. Laws and regulations should be conservative. This is particularly necessary with respect to chemical accident incidents. However, chemical accident damage is related to the chemicals’ physicochemical properties, whereas worker exposure levels are affected by the chemicals’ toxicity. The purpose of the regulations must be considered.

Appropriate regulatory concentrations should be established according to the regulation’s purpose, and concentrations should be set with consideration for the chemical’s physicochemical characteristics (specifically vapor pressure) and the reality of its use in the industrial field.

## Figures and Tables

**Table 1:** Physicochemical properties of selected chemical substances.

Property	Hydrogen Fluoride	Hydrogen Chloride	Hydrogen Peroxide	Aqueous Ammonia
CAS Number ^1^	7664-39-3	7647-01-0	7722-84-1	1336-21-6
Odor and color	Strong, irritating odor; colorless liquid or gas	Foul smelling odor; colorless liquid or gas	Odorless or weak ozone odor; colorless liquid	Strong, irritating odor; colorless liquid or gas
Molecular formula	HF	HCl	H_2_O_2_	NH_4_OH
Molecular weight	20.01	36.46	34.01	17.03
Melting point (°C)	−83.53	−33	−0.43	−77.7
Boiling point (°C)	19–20	65.6	152	36.35
Vapor density (g/L; air: 1)	0.92 (0 °C)	1.268 (25 °C)	1.17 (25 °C)	0.60 (25 °C)
Vapor pressure (mmHg)	917 (25 °C)	31,652 (20 °C)	1.97 (25 °C)	7510 (25 °C)
Purpose of use	Manufacture of refrigerants, disinfectants, fluoride raw materials, metal cleaners, disinfectants, etc.	Vinyl chloride polymer, production, oil well and steel pickling	Bleach, disinfectant, detergent, additive, oxidizing agent	Refrigerants and chemical industry applications (nitric acid, explosion production, synthetic fibers, fertilizer manufacturing, latex stabilization)

^1^ CAS Number: Chemical Abstracts Service Registry Number.

**Table 2:** Legal regulation of the subject chemical substances in the United States, European Union, Korea, China, and Japan.

Chemical	Regulatory Concentration in Each Country (Weight Percent)						
	United States (OSHA: PSM)	United States (EPA: RMP)	European Union (Seveso Directive)	Korea (Chemicals Control Act: Ministry of Environment)	Korea (PSM: Ministry of Employment and Labor)	China (Catalog of Hazardous Chemicals 2015)	Japan (ISHA ^1^)
Hydrogen fluoride	No regulation	50%	Comment ^3^	1%	1%	No regulation	5%
Hydrogen chloride	No regulation	37%	Comment ^3^	10%	10%	No regulation	1%
Hydrogen peroxide	52%	No criteria	Comment ^3^	35%/6% ^2^	52%	8%	1%
Aqueous ammonia	44%	20%	Comment ^3^	10%	10%	10%	0.1%

^1^ ISHA: Industrial Safety and Health Act. ^2^ 35%: Chemicals requiring preparation for accidents; 6%: Toxic chemicals. ^3^ Comment: EU Directive leaves it to the Member states for regulating the specifics so as they want it, as long as the goals as formulated in the Directive are achieved.

**Table 3:** Usage of chemicals in industry in Korea according to the 2017 annual report.

Chemical	Chemical Concentration, Weight Percent							
	1%–10%	10%–20%	20%–30%	30%–40%	40%–50%	50%–60%	60%–70%	>70%
	Annual Usage, Tons (Percent Overall Use)							
Hydrogen fluoride	-	-	-	286.85 (1.47%)	499.75 (2.57%)	18,637.77 (95.93%)	3.92 (0.02%)	-
Hydrogen chloride	27.49 (0.11%)	1,362.66 (5.47%)	32.78 (0.13%)	23,457.78 (94.28%)	-	-	-	-
Hydrogen peroxide	15.61 (0.02%)	460.13 (0.66%)	18.05 (0.03%)	58,947.69 (85.75%)	3,445.12 (5.01%)	29.6 (0.04%)	8,394.94 (12.21%)	0.32 (0.004%)
Aqueous ammonia	118.48 (0.49%)	510.21 (2.12%)	23,477.39 (97.39%)	-	-	-	-	-

**Table 4:** Vapor pressure by concentration of aqueous solution.

Hydrogen Fluoride		Hydrogen Chloride		Aqueous Ammonia		Hydrogen Peroxide	
Concentration (%)	Vapor Pressure (mmHg)	Concentration (%)	Vapor Pressure (mmHg)	Concentration (%)	Vapor Pressure (mmHg)	Concentration (%)	Vapor Pressure (mmHg)
37	4.5	20	0.3	1	0.1	5	0.01
38	4.0	21	0.5	2	0.2	10	0.04
39	6.3	22	0.7	3	0.3	15	0.05
40	9.9	23	1.2	4	0.5	20	0.07
41	12.5	24	1.5	5	0.7	25	0.08
42	14.7	25	2.1	6	0.9	30	0.09
43	17.8	26	3.2	7	1.0	35	0.15
44	20.1	27	4.2	8	1.2	40	0.23
45	21.3	28	7.1	9	1.5	45	0.30
46	25.0	29	10.3	10	1.8	50	0.38
47	28.0	30	15.1	11	2.0	55	0.41
48	32.6	31	25.2	12	2.4	60	0.53
49	40.5	32	32.5	13	2.0	65	0.68
50	47.0	33	40.1	14	3.0	70	0.90
51	50.1	34	68.5	15	3.3	75	1.01
52	52.3	35	86.1	16	3.5	80	1.13
53	55.1	36	142.0	17	3.9	85	1.28
54	57.3	37	176.4	18	4.2	90	1.50
55	61.7	38	277.0	19	4.7	95	1.73
56	65.9	39	356.0	20	5.5	100	1.88
57	73.5	40	515.0	21	6.0		
58	79.1	41	670.0	22	6.5		
59	85.1	42	900.0	23	7.0		
60	98.2			24	8.0		
61	105.1			25	8.5		
62	110.7			26	9.0		
63	118.1			27	10		
64	127.5			28	10.8		
65	135.6			29	11.5		
66	150.6			30	12.6		
67	161.2						
68	173.5						
69	186.4						
70	197.5						

**Table 5:** Pascal atmosphere stability.

Wind Speed (m/s)	Day			Night	
	Radiation Intensity				
	Strong	Moderate	Slight	Cloudy	Sunny
<2	A	A–B	B	F	F
2–3	A–B	B	C	E	F
3–5	B	B–C	C	D	E
5–6	C	C–D	D	D	D
>6	C	D	D	D	D

A: High instability, B: Instability, C: Slight instability, D: Neutral, E: Slight stability, F: High stability.

**Table 6:** Endpoint concentration of the studied chemicals.

Chemical	ERPG-2 Endpoint	AEGL-2 Endpoint
Hydrogen fluoride	20 ppm	24 ppm
Hydrogen chloride	20 ppm	22 ppm
Hydrogen peroxide	50 ppm	50 ppm ^1^
Aqueous ammonia	150 ppm	160 ppm

^1^ The PAC-2 value was applied for hydrogen peroxide because no corresponding AEGL-2 value exists.

**Table 7:** Damage radius of hydrogen fluoride by solution concentration.

Concentration (%)	KORA ERPG-2 Damage Radius (m)	KORA AEGL-2 Damage Radius (m)	ALOHA ERPG-2 Damage Radius (m)	ALOHA AEGL-2 Damage Radius (m)	PHAST ERPG-2 Damage Radius (m) ^1^	PHAST AEGL-2 Damage Radius (m) ^1^	RPM*Comp Damage Radius (m) ^2^
37	138.8	122.0	414.0	355	715.1	710.2	-
38	146.1	128.4	452.0	390	739.3	723.3	-
39	153.0	134.5	492.0	427.0	762.1	750.2	-
40	159.6	140.4	534.0	465.0	782.9	732.3	-
41	166.0	146.0	578.0	502.0	808.4	778.3	-
42	166.9	151.4	624.0	548.0	833.9	801.2	-
43	172.7	156.6	674.0	594.0	857.7	823.5	-
44	178.3	161.6	725.0	641.0	1375.3	1271.6	-
45	183.7	166.6	781.0	691.0	1399.6	1314.1	-
46	189.0	171.3	840.0	744.0	1422.9	1398.1	-
47	194.2	176.0	904.0	801.0	1447.6	1423.1	-
48	199.2	180.5	971.0	862.0	1468.9	1432.9	-
49	204.1	184.9	1013.0	926.0	1495.1	1457.2	-
50	208.9	189.3	1085.0	995.0	1517.6	1487.2	600.0
51	249.7	226.0	1203.0	1101.0	1540.2	1501.7	-
52	286.3	258.4	1291.0	1181.0	1564.5	1523.9	-
53	319.0	288.0	1307.0	1221.0	1585.1	1523.7	-
54	349.6	315.4	1411.0	1331.0	1632.0	1578.2	-
55	378.4	341.2	1582.0	1441.0	1655.2	1603.0	-
56	405.7	365.5	1691.0	1571.0	1678.1	1634.2	-
57	431.8	388.8	1825.0	1681.0	1698.8	1656.8	-
58	456.9	411.2	1901.0	1791.0	1722.3	1701.6	-
59	481.0	432.6	2003.0	1895.0	1742.5	1711.9	-
60	504.4	453.4	2283.0	2100.0	1767.2	1720.9	-
61	527.0	493.0	2395.0	2210.0	1785.5	1736.3	-
62	549.1	512.2	2450.0	2340.0	1811.2	1768.1	-
63	571.2	530.6	2610.0	2450.0	1830.0	1789.9	-
64	670.5	600.5	2800.0	2680.0	1854.1	1812.0	-
65	612.0	622.1	2910.0	2790.0	1874.0	1835.8	-
66	718.8	643.2	3080.0	2915.0	1896.6	1857.5	-
67	651.8	584.0	3270.0	3130.0	1917.2	1885.1	-
68	671.1	600.1	3510.0	3310.0	1939.3	1903.2	-
69	690.2	618.0	3720.0	3560.0	2017.1	1956.2	-
70	708.8	634.5	3910.0	3790.0	-	-	1900.0

^1^ PHAST does not support modeling for aqueous solutions of 70%. ^2^ RMP*Comp supports modeling only for concentrations of 50% and 70%.

**Table 8:** Damage radius of hydrogen chloride by solution concentration.

Concentration (%)	KORA ERPG-2 Damage Radius (m)	KORA AEGL-2 Damage Radius (m)	ALOHA ERPG-2 Damage Radius (m)	ALOHA AEGL-2 Damage Radius (m)	PHAST ERPG-2 Damage Radius (m)	PHAST AEGL-2 Damage Radius (m)	RMP*Comp Damage Radius (m) ^1^
20	22.4	20.0	57.0	56.0	250.8	246.1	-
21	28.9	26.0	68.0	68.0	253.5	246.5	-
22	34.1	30.9	83.0	83.0	255.6	252.1	-
23	43.8	39.7	108.0	106.0	259.2	259.1	-
24	51.7	46.9	155.0	149.0	276.2	270.9	-
25	65.5	59.5	234.0	222.0	311.2	297.5	-
26	76.9	69.9	339.0	325.0	336.8	323.0	-
27	98.3	89.3	464.0	446.0	367.5	351.5	-
28	116.0	105.4	609.0	587.0	401.7	384.3	-
29	147.2	133.5	777.0	749.0	438.8	420.7	-
30	173.3	157.1	981.0	945.0	486.7	466.7	-
31	221.2	200.3	1230.0	1200.0	553.0	531.9	-
32	261.9	236.9	1910.0	1800.0	621.8	597.3	-
33	334.2	301.6	2250.0	2100.0	633.3	606.5	-
34	396.5	357.3	2680.0	2500.0	640.6	621.0	-
35	507.6	456.3	2910.0	2800.0	675.9	622.8	-
36	604.9	544.1	3200.0	3100.0	702.1	649.3	-
37	764.9	683.9	3700.0	3600.0	709.3	682.4	1800.0
38	908.8	810.4	4100.0	3900.0	729.0	721.3	2000.0
39	1141.0	1013.7	4300.0	4200.0	1451.7	1376.2	-
40	1354.1	1197.6	4600.0	4500.0	1484.2	1381.2	-
41	1676.3	1497.7	4900.0	4700.0	1574.2	1389.6	-
42	1981.1	1741.9	6000.0	5700.0	1678.1	1428.7	-

^1^ RMP*Comp supports modeling only for concentrations of 37% and 38%.

**Table 9:** Damage radius of hydrogen peroxide by solution concentration.

Concentration (%)	KORA ERPG-2 Damage Radius (m)	KORA AEGL-2 Damage Radius (m)	ALOHA ERPG-2 Damage Radius (m)	ALOHA AEGL-2 Damage Radius (m)	PHAST ERPG-2 Damage Radius (m)	PHAST AEGL-2 Damage Radius (m)	RMP*Comp Damage Radius (m) ^1^
5	17.6	17.6	152.0	152.0	187.1	187.1	-
10	17.5	17.5	151.0	151.0	244.2	244.2	-
15	17.6	17.6	155.0	155.0	274.2	274.2	-
20	17.3	17.3	145.0	145.0	304.1	304.1	-
25	17.3	17.3	142.0	142.0	324.0	324.0	-
30	17.2	17.2	135.0	135.0	355.0	355.0	-
35	17.1	17.1	130.0	130.0	378.6	378.6	-
40	18.6	18.6	176.0	176.0	406.4	406.4	-
45	20.1	20.1	211.0	211.0	427.6	427.6	-
50	21.5	21.5	223.0	223.0	447.0	447.0	-
55	21.5	21.5	228.0	228.0	469.2	469.2	-
60	22.8	22.8	231.0	231.0	488.5	488.5	-
65	24.1	24.1	252.0	252.0	503.4	503.4	-
70	26.4	26.4	270.0	270.0	517.6	517.6	-
75	27.5	27.5	278.0	278.0	540.2	540.2	-
80	28.5	28.5	290.0	290.0	601.3	601.3	-
85	29.5	29.5	301.0	301.0	546.8	546.8	-
90	30.5	30.5	305.0	305.0	372.6	372.6	-
95	31.4	31.4	315.0	315.0	294.1	294.1	-
100	32.4	32.4	323.0	323.0	210.4	210.4	-

^1^ RMP*Comp does not currently support modeling for hydrogen peroxide.

**Table 10:** Damage radius of aqueous ammonia by concentration.

Concentration (%)	KORA ERPG-2 Damage Radius (m)	KORA AEGL-2 Damage Radius (m)	ALOHA ERPG-2 Damage Radius (m)	ALOHA AEGL-2 Damage Radius (m)	PHAST ERPG-2 Damage Radius (m)	PHAST AEGL-2 Damage Radius (m)	RMP*Comp Damage Radius (m) ^1^
1	62.0	59.9	446.0	426.0	259.8	253.9	-
2	89.4	86.4	485.0	467.0	379.6	373.6	-
3	110.8	112.4	557.0	526.0	443.7	435.4	-
4	129.3	124.9	625.0	600.0	479.5	476.1	-
5	145.1	140.4	663.0	648.0	523.0	507.8	-
6	158.9	153.2	738.0	714.0	564.1	551.8	-
7	171.6	165.2	802.0	777.0	598.2	581.7	-
8	183.7	177.4	871.0	844.0	641.8	622.0	-
9	195.3	188.6	946.0	917.0	675.0	657.4	-
10	208.9	201.7	1000.0	944.0	712.6	685.2	-
11	224.2	212.6	1150.0	1100.0	751.2	725.4	-
12	238.8	230.5	1210.0	1198.0	824.2	759.5	-
13	252.9	244.1	1270.0	1220.0	859.9	789.9	-
14	266.5	257.2	1410.0	1380.0	894.4	823.8	-
15	284.6	274.0	1480.0	1420.0	932.7	862.2	-
16	303.9	293.1	1570.0	1510.0	969.0	892.4	-
17	322.5	311.0	1610.0	1580.0	1004.8	931.1	-
18	340.4	328.2	1700.0	1630.0	1040.8	964.1	-
19	357.9	345.0	1780.0	1740.0	1076.2	994.4	-
20	382.5	368.7	1880.0	1810.0	1112.7	1032.9	300.0
21	407.1	392.3	1950.0	1890.0	1150.5	1064.6	-
22	430.9	415.2	2000.0	1910.0	1181.4	1098.2	-
23	454.2	437.5	2060.0	2000.0	1219.6	1131.2	-
24	477.4	459.8	2170.0	2100.0	1255.9	1164.5	400.0
25	509.7	490.7	2310.0	2190.0	1290.4	1197.3	-
26	541.0	519.6	2400.0	2230.0	1327.6	1233.3	-
27	571.5	550.0	2500.0	2340.0	1360.6	1265.5	-
28	601.3	578.6	2600.0	2450.0	1393.6	1295.8	-
29	632.6	608.5	2700.0	2570.0	1432.2	1329.7	-
30	672.1	646.3	2900.0	2710.0	1465.1	1363.7	500.0

^1^ RMP*Comp supports modeling only for concentrations of 20%, 24%, and 30%.

**Table 11:** Damage radius ratio of concentration commonly used in industry to concentration regulated by the Chemicals Control Act.

Chemical	KORA		ALOHA		PHAST	
	ERPG-2	AEGL-2	ERPG-2	AEGL-2	ERPG-2	AEGL-2
Hydrogen fluoride (55% vs. 37%)	2.76	2.79	3.82	4.05	2.31	2.25
Hydrogen chloride (35% vs. 20%)	22.66	22.81	51.05	50.01	2.69	2.53
Hydrogen peroxide (35% vs. 6%)	0.97	0.97	0.85	0.85	2.02	2.02
Aqueous ammonia (25% vs. 10%)	2.44	2.43	2.31	2.32	1.81	1.74
